# Prediction of the pathological subtypes by intraoperative frozen section for patients with cT1N0M0 invasive lung adenocarcinoma (ECTOP-1015): a prospective multicenter study

**DOI:** 10.1097/JS9.0000000000001667

**Published:** 2024-05-23

**Authors:** Zichen Fu, Xuxia Shen, Chaoqiang Deng, Hang Cao, Yan Jin, Qiang Zheng, Yongguo Yang, Bin Qian, Chunyan Yuan, Weihua Wang, Lei Zhang, Qingping Song, Shuying Zuo, Junjie Ma, Shuqing You, Senzhong Zheng, Qingli Gao, Guangli Su, Yang Zhang, Fangqiu Fu, Haiquan Chen, Yuan Li

**Affiliations:** a Department of Thoracic Surgery, Fudan University Shanghai Cancer Center; b Department of Pathology, Fudan University Shanghai Cancer Center; c Department of Oncology, Shanghai Medical College, Fudan University; d Institute of Thoracic Oncology, Fudan University, Shanghai; e Department of Pathology, Minhang Hospital & School of Pharmacy, Fudan University; f Department of Thoracic Surgery, Minhang Hospital & School of Pharmacy, Fudan University, Shanghai; g Department of Pathology, Jiangdu People’s Hospital Affiliated to Medical College of Yangzhou University; h Department of Thoracic Surgery, Jiangdu People’s Hospital Affiliated to Medical College of Yangzhou University, Jiangsu; i Department of Pathology, Liaocheng Cancer Hospital; j Department of Thoracic Surgery, Liaocheng Cancer Hospital; k Department of Pathology, Liaocheng Second People’s Hospital; l Department of Thoracic Surgery, Liaocheng Second People’s Hospital; m Department of Pathology, Guanxian People’s Hospital; n Department of Thoracic Surgery, Guanxian People’s Hospital, Shangdong; o Department of Pathology, Taizhou First People’s Hospital; p Department of Thoracic Surgery, Taizhou First People’s Hospital, Zhejiang, People’s Republic of China

**Keywords:** intraoperative frozen section, lung invasive adenocarcinoma, nonsmall cell lung cancer, pathological subtype

## Abstract

**Background:**

This study aims to assess the diagnostic accuracy of the intraoperative frozen section (FS) in determining the pathological subtypes among patients diagnosed with cT1N0M0 invasive lung adenocarcinoma.

**Materials and methods:**

This was a prospective, multicenter (seven centers in China) clinical trial of Eastern Cooperative Thoracic Oncology Projects (ECTOP-1015). Patients with cT1N0M0 invasive lung adenocarcinoma were enrolled in the study. Pathological images obtained from FS and final pathology (FP) were reviewed by at least two pathologists. The primary endpoint was the concordance between FS and FP diagnoses. The interobserver agreement for identifying pathological subtypes on FS was evaluated among three pathologists.

**Results:**

A total of 935 patients were enrolled. The best sensitivity of diagnosing the predominant subtype was 78.2% in the evaluation of the acinar pattern. The presence of an acinar pattern diagnosed by FS was an independent factor for the concordance between FS and FP (*P*=0.007, 95% confidence interval: 2.332–4.736). Patients with tumor size >2 cm measured by pathology showed a better concordance rate for the predominant subtype (81.6% vs. 74.6%, *P*=0.023). The presence of radiological ground glass opacity component did not affect the diagnosis accuracy of FS for the predominant subtype (concordance rate: 76.4% vs. 75.2%, *P*=0.687). Patients with ground glass opacity component showed better accuracy of the identification in the presence of lepidic pattern-predominant adenocarcinoma (82.1% vs. 71.0%, *P*=0.026). Substantial agreement between the FS diagnosis from three pathologists for the predominant pathological pattern was revealed with κ=0.846.

**Conclusions:**

This is the largest prospective trial evaluating FS diagnosing pathological subtype in cT1N0M0 invasive lung adenocarcinoma. A favorable concordance in the assessment of the pathological subtypes between FS and FP was observed, indicating the feasibility of utilizing accurate intraoperative pathological diagnoses from FS in guiding surgical strategies. A combination of radiology could improve the precision of FS.

## Introduction

Highlights

- The largest cohort for frozen section (FS) diagnosing pathological subtype in cT1N0M0 invasive LUAD.
- Favorable agreement between FS and final pathology (FP) for pathological subtype was observed.
- FS could guide surgery by the diagnosis of pathological subtype.

Lung adenocarcinoma is currently the most prevalent histological type of lung malignancy, accounting for over 40% of all lung cancer cases^1–4^. In 2011, the International Association for the Study of Lung Cancer/ American Thoracic Society/European Respiratory Society released the classification system for lung adenocarcinoma^5^, later adopted by the WHO^6,7^. Invasive nonmucinous lung adenocarcinoma can be primarily categorized into five pathological subtypes, including lepidic, acinar, papillary, solid, and micropapillary patterns. Among these, the lepidic pattern is associated with improved survival, while solid and micropapillary patterns indicate poorer outcomes^8–10^. Typically, the adenocarcinoma subtype is determined in the FP following surgery, failing to guide surgical procedures.

The JCOG0802^11^ and CALGB140503^12^ trials have concluded that sublobar resection is adequate for treating peripheral nonsmall cell lung cancer with a size of less than 2 cm. Sublobar resection offers various advantages, including the preservation of more normal lung tissue, improved perioperative outcomes, and increased feasibility for undergoing surgical resection for second primary lung cancer when compared to lobectomy^11,13,14^. Sublobar resection is recognized as a significant surgical procedure in the treatment of early-stage nonsmall cell lung cancer. Tumor size and consolidation-to-tumor ratio are commonly utilized parameters in determining the suitability for sublobar resection. Previously, we proposed that the intraoperative FS was an effective tool for guiding sublobar resection^15^. Some studies reported that patients with low-risk adenocarcinoma subtypes could be candidates for sublobar resection, while high-risk adenocarcinoma may require a larger extent of surgical resection^16,17^. Although FS examinations can assist in making intraoperative pathological diagnoses regarding overall histology^15,18^, it remains uncertain whether adenocarcinoma subtypes can be accurately assessed via FS.

In this study, we conducted a prospective multicenter study to evaluate the accuracy of intraoperative FS in identifying the pathological subtypes of early-stage invasive lung adenocarcinoma patients. Additionally, we performed subgroup analysis to explore variations in the accuracy of FS among patients with distinct clinicopathological characteristics.

## Methods

### Study design

**Figure 1:**
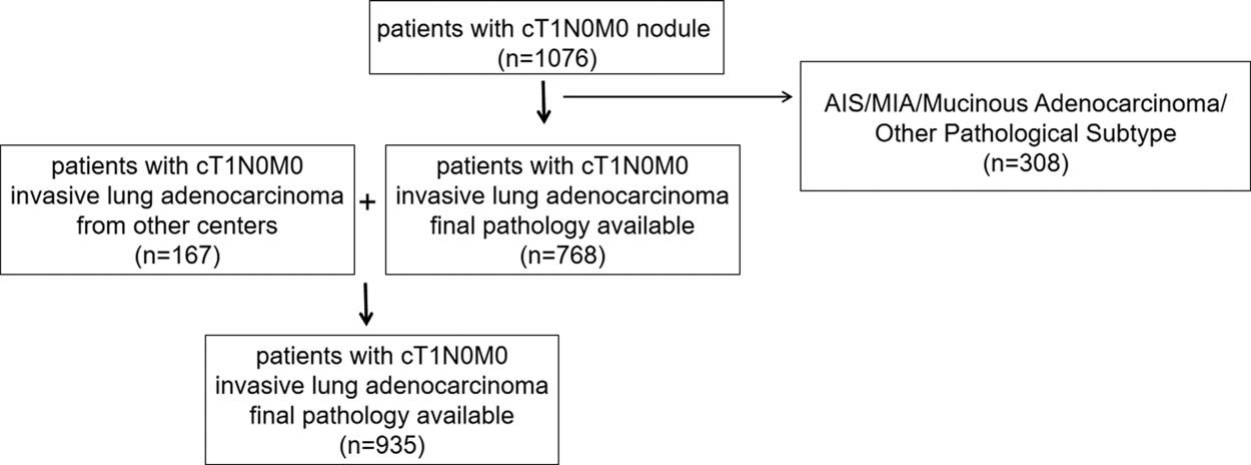
The flowchart of enrollment of patients.

This study was designed as a prospective, multicenter clinical trial conducted across seven centers in China under the Eastern Cooperative Thoracic Oncology Projects (ECTOP-1015). Patients were eligible for enrollment if they met the following inclusion criteria: cT1N0M0, cytologically or histologically confirmed primary lung adenocarcinoma, and undergoing radical surgical resection. Patient recruitment started from July 2022 to August 2023. The exclusion criteria included: cytologically or histologically confirmed benign disease, adenocarcinoma in situ (AIS)/minimally invasive adenocarcinoma (MIA), malignancy other than lung adenocarcinoma, or invasive mucinous adenocarcinoma; history of other malignant tumors; prior neoadjuvant therapy including radiotherapy or chemotherapy; and presence of multiple pulmonary nodules unless pathologically diagnosed as AIS/MIA for the remaining nodules. The flowchart of this study is depicted in Figure 1. The primary endpoint of this study was the concordance between intraoperative frozen pathology and postoperative paraffin FP in diagnosing pathological subtypes. The concordance between FS and FP was defined as identical pathological subtypes diagnosed by intraoperative FS and postoperative FP. If *n* represents the number of all enrolled cases, and *m* represents the number of patients with identical pathological subtypes in FS and FP, the concordance rate is defined as *m*/*n*.

### Criteria for preoperative clinical staging and surgery

All enrolled patients underwent a routine evaluation for clinical staging, including thin-layer (1 mm) enhanced computed tomography (CT) scans. Clinical staging was mainly assessed through chest CT scans. Specifically, cT1 was characterized by tumors with the maximum diameter of ≤3 cm radiologically, absence of obvious pleural invasion, no main bronchial invasion/pulmonary atelectasis/obstructive pneumonia, no invasion of chest wall/nerve/pericardium, and no isolated malignant nodule within the same lobe or in a different lobe on the same side of the lung. If the size of intrapulmonary, interlobular, hilar, and mediastinal lymph nodes measured less than 10 mm on CT scans, they were categorized as N0. The surgical procedure was determined comprehensively based on several factors, including nodule size, consolidation-to-tumor ratio, and location within the lung. The final surgical procedure should be regarded as radical oncologically in accordance with current medical knowledge and practices.

### Pathological evaluation

Frozen pathology samples were obtained from the largest diameter of the tumor. Diagnosis of postoperative tumor paraffin pathology diagnosis and FS were made according to the 2021 version of the WHO classification criteria^7^. The histological types include AIS, MIA, and invasive adenocarcinoma, which was further categorized into lepidic pattern, acinar pattern, papillary pattern, micropapillary pattern, solid pattern, and other uncommon patterns. The predominant pathological subtype was defined as the subtype with the largest percentage without a strict threshold requirement (not necessarily 50% or higher). Ten pathologists underwent either in-person (a microscope) or virtual training sessions to standardize their diagnostic procedures. An interobserver consistency assessment was conducted among the diagnoses provided by three pathologists to ensure consistency and credibility.

### Statistical analysis

The concordance between intraoperative FS and final paraffin pathology, with regard to the predominant pathological pattern and presence of certain pathological patterns, was evaluated by kappa statistics and correlation analysis. Fleiss’ kappa coefficients were utilized to assess the interobserver agreement between three pathologists in the pathological diagnosis by intraoperative FS. The degree of agreement was interpreted as follows: slight agreement (κ=0–0.20), fair agreement (κ=0.21–0.40), moderate agreement (κ=0.41–0.60), substantial agreement (κ=0.61–0.80), and almost perfect agreement (κ≥0.81). Logistic regression was applied to examine the factors contributing to discrepancies between intraoperative FS and postoperative FP. A *P* value less than 0.05 in this study was considered to be a significant difference. Statistical analyses were performed using SPSS software (version 25.0; IBM).

This prospective, multicenter clinical trial was carried out in seven centers. This study was conducted in accordance with the Declaration of Helsinki (as revised in 2013). All enrolled patients provided written informed consent. This work has been reported in line with the STROCSS criteria^19^, Supplemental Digital Content 1, http://links.lww.com/JS9/C659.

## Results

### Characteristics of patients

The prospective enrollment comprised 935 patients diagnosed with cT1N0M0 nonmucinous invasive lung adenocarcinoma. Among them, 374 (40.0%) were males, and 561 (60.0%) were females, with a median age of 62 years. The majority of patients were nonsmokers, accounting for 67.8% of the cohort. Seventy-five (8.0%) patients were diagnosed with cT1a, 509 (54.4%) with cT1b, and 351 (37.5%) with cT1c according to the eighth TNM grading system. Pathological assessments revealed that 126 patients (13.5%) were diagnosed with pT2. Seventy-eight patients (8.3%) exhibited pathological lymph node involvement, with 36 patients (3.9%) diagnosed as pN1 and 42 (4.4%) as pN2. The characteristics of all included patients were summarized in Table 1.

**Table 1:** The clinicopathological characteristics of enrolled patients.

Characteristics	*N*=935
Age, median (range)	62 (29–84)
Gender, *n* (%)	
Male	374 (40.0)
Female	561 (60.0)
Smoking history, *n* (%)	
Ever smoker	301 (32.2)
Never smoker	634 (67.8)
Cancer description	
Median size (cm) (minimum–maximum)	1.6 (0.5–4.8)
Range of resection, *n* (%)	
PEN	1 (0.1)
LOB	410 (43.9)
SEG	327 (35.0)
WED	197 (21.1)
pT staging, *n* (%)	
pT1a	116 (12.4)
pT1b	509 (54.4)
pT1c	184 (19.7)
pT2a	121 (12.9)
pT2b	5 (0.5)
pN staging, *n* (%)	
pN0	857 (91.7)
pN1	36 (3.9)
pN2	42 (4.5)
pTNM staging, *n* (%)	
IA	763 (81.6)
IB	90 (9.6)
IIA	4 (0.4)
IIB	36 (3.8)
IIIA	42 (4.4)
cT staging, *n* (%)	
cT1a	82 (8.8)
cT1b	502 (53.7)
cT1c	351 (37.5)

LOB, lobectomy; PEN, pneumonectomy; SEG, segmentectomy; TNM staging, tumor node metastasis staging; WED, wedge resection.

### Diagnoses of predominant pathological subtype by frozen section and final pathology

Within the total cohort of 935 enrolled patients, the FP revealed the following predominant patterns: 93 individuals (9.9%) classified as lepidic pattern-predominant adenocarcinoma (LPA), 617 (66.0%) as acinar pattern-predominant adenocarcinoma (APA), 146 (15.6%) as papillary pattern-predominant adenocarcinoma (PPA), 43 (4.5%) as solid pattern-predominant adenocarcinoma (SPA), and 16 (1.7%) as micropapillary pattern-predominant adenocarcinoma (MPA). Additionally, 20 (2.1%) patients observed that acinar and papillary patterns occupied the majority of the lesions, with both patterns accounting for an equal proportion. Diagnoses based on intraoperative FS categorized patients as follows: 98 (10.5%) as LPA, 556 (59.4%) as APA, 165 (17.6%) PPA, 55 (5.8%) as SPA, and five (0.5%) as MPA. Fifty-six (5.9%) patients were identified as having acinar/papillary pattern predominant, with both subtypes having an equal percentage.

The total concordance rate of the cohort was 76.1%. The sensitivity of diagnosing LPA, APA, PPA, SPA, and MPA were 61.2%, 79.2%, 65.7%, 72.0%, and 18.7%, respectively (*P*<0.001). Additionally, the specificity of diagnosing LPA, APA, PPA, SPA, and MPA were 95.1%, 68.8%, 90.4%, 97.3%, and 99.8%, respectively. The concordance rates between FS and FP for the diagnosis of LPA, APA, PPA, SPA, and MPA were 91.8%, 72.0%, 87.4%, 96.1%, and 98.4%, respectively (Table 2).

**Table 2:** The concordance rate between frozen section and final pathology for the diagnosis of predominant pathological subtype and presence of certain subtype.

	Category	Sensitivity	Specificity	PPV/precision	NPV	Accuracy
Predominant subtype	L	61.2%	95.1%	58.1%	95.7%	91.8%
	A	79.2%	68.8%	86.3%	72.8%	72.0%
	P	65.7%	90.4%	56.9%	94.5%	87.4%
	S	72.0%	97.3%	56.3%	98.6%	96.1%
	M	18.7%	99.8%	60.0%	98.6%	98.4%
Presence of certain subtypes	L	79.8%	62.6%	68.0%	75.7%	71.2%
	A	84.3%	58.4%	95.7%	25.1%	82.2%
	P	62.4%	77.7%	80.0%	59.3%	68.8%
	S	65.1%	91.1%	53.8%	94.3%	87.5%
	M	45.5%	89.0%	61.9%	80.7%	76.8%
	CGP	60.1%	77.2%	52.1%	82.4%	72.2%

A, acinar pattern; CGP, complex glandular pattern; L, lepidic pattern; M, micropapillary pattern; NPV, negative predictive value; P, papillary pattern; PPV, positive predictive value; S, solid pattern.

### Diagnoses of presence and absence of certain pathological subtypes by frozen section and final pathology

According to paraffin FP, the numbers of patients diagnosed with lepidic, acinar, papillary, solid, micropapillary patterns, and complex glands pattern (CGP) were 457 (48.8%), 858 (91.7%), 549 (58.7%), 129 (13.7%), 263 (28.3%), and 276 (29.5%), respectively. According to intraoperative FS, the numbers of patients diagnosed with lepidic, acinar, papillary, solid, micropapillary patterns, and CGP were 548 (58.6%), 756 (80.8%), 429 (45.8%), 156 (16.6%), 189 (20.2%), and 309 (33.0%), respectively.

FS analysis demonstrated its highest sensitivity in detecting the presence of the acinar pattern (87.5%). Conversely, the lowest sensitivity was observed in identifying the presence of the micropapillary pattern (45.5%). The highest and lowest specificity was observed with the solid pattern (91.1%) and acinar patterns (58.4%), respectively. The concordance rates between FS and FP for the diagnosis of lepidic, acinar, papillary, solid, micropapillary patterns, and CGP were 71.2%, 82.2%, 68.8%, 87.5%, 76.8%, and 72.2%, respectively (Table 2).

### Performance of frozen section for diagnosing pathological subtypes in subgroups

Patients with pathological tumor size greater than 2 cm had a significantly higher concordance rate of FS and FP for the diagnosis of the predominant pathological subtype compared to those with tumor size less than 2 cm (81.6% vs. 74.6%, *P*=0.023). However, when tumor size was assessed by radiology, both groups exhibited similar concordance rates (75.8% vs. 76.6%, *P*=0.796). Supplementary Table 1, Supplemental Digital Content 2, http://links.lww.com/JS9/C660 provides the concordance rates between FS and FP for diagnosing pathological subtypes stratified by pathological or radiological tumor size. Regarding the evaluation of the presence or absence of certain pathological subtypes, better performance of FS was observed in patients with the size greater than 2 cm measured compared to those with the size of less than 2 cm, regardless of measurement by pathology or radiology (Table 3). The concordance rates for the diagnosis of the presence or absence of certain pathological subtypes between FS and FP, stratified by pathological and radiological size, are shown in Table 3.

**Table 3:** The concordance rate between frozen section and final pathology among patients with tumor size >2 cm and ≤2 cm, measured by pathology and radiology, for the diagnosis of the presence or absence of certain pathological subtypes.

	>2 cm						≤2 cm						
	Category	Sensitivity	Specificity	PPV/precision	NPV	Accuracy	Sensitivity	Specificity	PPV/precision	NPV	Accuracy	*P* (sensitivity)	*P* (specificity)
Radiology	L	84.6%	63.9%	61.7%	85.8%	72.4%	78.3%	61.1%	70.1%	70.8%	70.4%	0.118	0.696
	A	85.3%	55.6%	94.3%	39.0%	82.3%	83.7%	61.0%	96.6%	22.1%	82.2%	0.431	0.630
	P	60.0%	76.4%	81.2%	54.8%	65.8%	64.1%	80.4%	78.3%	64.1%	70.5%	0.603	0.675
	S	65.1%	88.8%	57.3%	91.7%	84.3%	65.0%	92.3%	50.6%	95.6%	89.4%	0.993	0.091
	M	46.3%	85.6%	67.0%	71.6%	70.4%	42.5%	91.0%	56.8%	85.1%	80.5%	0.535	**0.033**
	CGP	64.8%	74.5%	59.6%	78.1%	70.7%	55.8%	80.2%	48.2%	84.6%	74.1%	0.126	0.132
Pathology	L	83.6%	67.4%	56.4%	89.1%	72.9%	79.3%	58.5%	71.2%	68.5%	72.2%	0.329	**0.040**
	A	85.9%	59.4%	94.8%	32.6%	83.2%	83.6%	57.8%	96.7%	21.7%	81.8%	0.456	0.899
	P	62.6%	76.9%	84.3%	51.4%	67.4%	62.3%	78.0%	77.0%	63.8%	69.4%	0.940	0.819
	S	65.7%	86.7%	58.9%	89.6%	81.9%	64.4%	92.9%	48.7%	96.1%	90.2%	0.877	**0.004**
	M	44.9%	84.3%	69.6%	65.6%	66.8%	44.0%	91.0%	55.0%	86.6%	81.6%	0.880	**0.014**
	CGP	63.5%	75.8%	63.9%	71.2%	70.3%	56.8%	87.7%	45.6%	82.9%	80.8%	0.258	**0.014**

Bold values indicate statistically significant (*P*<0.05).

A, acinar pattern; CGP, complex glandular pattern; L, lepidic pattern; M, micropapillary pattern; NPV, negative predictive value; P, papillary pattern; PPV, positive predictive value; S, solid pattern.

Patients with ground-glass opacity component in radiology showed a similar concordance rate between FS and FP for the diagnoses of predominant pathological subtype compared to solid lesions (76.4% vs. 75.2%, *P*=0.687). However, patients with ground glass opacity component exhibited better sensitivity for the identification of the presence of lepidic pattern compared to radiological solid patients (82.1% vs. 71.0%, *P*=0.026) (Table 4). Similar sensitivity between the two subgroups was detected regarding the presence of other pathological subtypes, including acinar, papillary, solid, micropapillary patterns, and CGP (84.4% vs. 84.1%, *P*=0.841; 63.9% vs. 59.7%, *P*=0.337; 68.4% vs. 63.7%, *P*=0.611; 46.7% vs. 42.8%, *P*=0.527; 60.0% vs. 60.2%, *P*=0.969 for acinar, papillary, solid, micropapillary patterns, and CGP, respectively) (Table 4).

**Table 4:** The concordance rate between frozen section and final pathology among patients with different radiology for the diagnosis of predominant pathological subtype and presence of certain subtype.

	Lesions with GGO component						Solid lesions						
	Category	Sensitivity	Specificity	PPV/precision	NPV	Accuracy	Sensitivity	Specificity	PPV/precision	NPV	Accuracy	*P* (sensitivity)	*P* (specificity)
Predominant subtype	L	63.2%	94.1%	64.0%	93.8%	88.3%	66.7%	98.4%	28.6%	99.7%	98.1%	0.903	**0.002**
	A	80.2%	73.8%	86.7%	63.7%	78.1%	80.0%	73.9%	87.3%	68.0%	78.2%	0.975	0.855
	P	69.6%	90.1%	56.4%	94.2%	86.9%	77.2%	90.6%	58.6%	95.8%	88.6%	0.357	0.970
	S	75.0%	98.9%	46.2%	99.7%	98.5%	71.4%	94.0%	59.5%	96.4%	91.6%	0.839	**<0.001**
	M	33.3%	99.8%	66.7%	99.3%	99.2%	10.0%	99.7%	50.0%	97.2%	96.9%	0.247	0.626
Presence of certain subtype	L	82.1%	51.3%	73.1%	63.8%	70.4%	71.0%	73.0%	45.0%	99.0%	72.5%	**0.026**	**<0.001**
	A	84.4%	56.3%	96.3%	23.7%	82.3%	84.1%	62.1%	95.3%	27.0%	83.5%	0.841	0.616
	P	63.9%	76.9%	78.5%	61.7%	69.4%	59.7%	79.4%	80.6%	56.9%	67.5%	0.337	0.589
	S	68.4%	93.9%	42.6%	97.8%	92.4%	63.7%	83.8%	61.1%	85.3%	78.1%	0.611	**<0.001**
	M	46.7%	92.1%	56.0%	88.9%	84.1%	42.8%	80.7%	67.3%	60.4%	62.5%	0.527	**<0.001**
	CGP	60.0%	82.9%	42.0%	91.0%	78.5%	60.2%	74.5%	64.8%	68.9%	66.9%	0.969	**<0.001**

Bold values indicate statistically significant (*P*<0.05).

A, acinar pattern; CGP, complex glandular pattern; GGO, ground glass opacity; L, lepidic pattern; M, micropapillary pattern; NPV, negative predictive value; P, papillary pattern; PPV, positive predictive value; S, solid pattern.

### Misdiagnosis of pathological subtype by frozen section

Two hundred thirteen patients exhibited a discrepancy between FS and FP upon the predominant pathological subtype (Table 5). Patients with a discrepancy between FS and FP were observed to have a higher prevalence of lepidic predominant lesions (19.2% vs. 8.7%, *P*<0.001) and a lower prevalence of acinar predominant pattern (35.2% vs. 71.8%, *P*<0.001) according to FS. As for the presence of subtypes, patients with the discrepancy between FS and FP were associated with a lower prevalence of acinar pattern (62.4% vs. 85.2%, *P*<0.001) and CGP (25.4% vs. 35.3%, *P*=0.011) according to FS. Further analysis also indicated that the presence of an acinar pattern diagnosed by FS was an independent factor for the concordance between FS and FP [*P*<0.001, odds ratio (OR)=0.309, 95% confidence interval (CI): 0.216–0.442). (Supplementary Table 2, Supplemental Digital Content 3, http://links.lww.com/JS9/C661).

**Table 5:** The correlation between frozen section diagnosis and clinicopathologic features of the patients.

Characteristics	Diagnosis with concordance (*N*=657)	Diagnosis with discrepancy (*N*=213)	*P*
Age, median (range)	62 (29–75)	62 (30–81)	0.434
Gender, *n* (%)			0.662
Male	264 (40.2)	82 (38.5)	
Female	393 (59.8)	131 (61.5)	
Smoking history, *n* (%)			0.628
Ever smoker	180 (27.4)	62 (29.1)	
Never smoker	477 (72.6)	151 (70.9)	
Median size (range), cm	1.6 (0.5–4.8)	1.65 (0.5–4.0)	
Range of resection, *n* (%)			0.019
LOB	306 (46.6)	76 (35.7)	
SEG	219 (33.3)	83 (39.0)	
WED	132 (20.1)	54 (25.4)	
pT staging, *n* (%)			0.122
pT1a	87 (13.2)	22 (10.3)	
pT1b	345 (52.5)	129 (60.6)	
pT1c	138 (21.0)	33 (15.5)	
pT2a	82 (12.5)	29 (13.6)	
pT2b	5 (0.7)	0	
pN staging, *n* (%)			0.727
pN0	604 (91.9)	197 (92.5)	
pN1	24 (3.7)	9 (4.2)	
pN2	29 (4.4)	7 (3.3)	
LVI+	91 (15.0)	40 (19.1)	0.081
VPI+	90 (13.5)	26 (12.5)	0.578
STAS+	178 (27.0)	55 (26.2)	0.716
LVI+ (FS)	79 (12.0)	18 (8.5)	0.150
VPI+ (FS)	92 (14.0)	24 (11.2)	0.307
STAS+ (FS)	122 (18.6)	21 (9.9)	0.438
Predominant subtypes (FP)			＜0.001
L	57 (8.7)	27 (12.7)	
A	472 (71.8)	122 (57.3)	
P	94 (14.3)	39 (18.3)	
S	31 (4.7)	12 (5.6)	
M	3 (0.5)	13 (6.1)	
Predominant subtypes (FS)			＜0.001
L	57 (8.7)	41 (19.2)	
A	472 (71.8)	75 (35.2)	
P	94 (14.3)	41 (19.2)	
S	31 (4.7)	24 (11.3)	
M	3 (0.5)	2 (0.9)	
Presence of subtypes (FP)			
L	325 (49.5)	111 (52.1)	0.502
A	601 (91.5)	197 (92.5)	0.641
P	355 (54.0)	140 (65.7)	0.003
S	91 (13.9)	35 (16.4)	0.352
M	179 (27.2)	60 (28.2)	0.793
CGP	184 (28.0)	72 (33.8)	＜0.001
Presence of subtypes (FS)			
L	386 (58.8)	138 (64.8)	0.118
A	560 (85.2)	133 (62.4)	＜0.001
P	271 (41.2)	104 (48.8)	0.052
S	103 (15.7)	43 (20.2)	0.126
M	132 (20.1)	39 (18.3)	0.635
CGP	232 (35.3)	54 (25.4)	0.011

A, acinar pattern; CGP, complex glandular pattern; FP, final pathology; FS, frozen section; L, lepidic pattern; LVI, lympho-vascular invasion; M, micropapillary pattern; P, papillary pattern; S, solid pattern; STAS, spread through air space; VPI, visceral pleural invasion.

### Interobserver agreement of frozen section for diagnosis of pathological subtype

Three pulmonary pathologists independently reviewed FS slides from the first 52 cases to investigate the interobserver agreement concerning the diagnoses of pathological subtypes. Remarkable consistency upon the predominant pathological pattern of lesions was revealed with a concordance of 76.1% (κ=0.846), indicating substantial agreement. The κ values for agreement among three pathologists regarding the presence or absence of lepidic, acinar, papillary, solid, micropapillary patterns, and CGP were 0.814 (95% CI: 0.809–0.819), 0.949 (95% CI: 0.944–0.954), 0.708 (95% CI: 0.703–0.713), 0.829 (95% CI: 0.824–0.834), 0.531 (95% CI: 0.526–0.536), and 0.810 (95% CI: 0.804–0.815), respectively (Table 6). Substantial agreement was evident in most pathological subtypes except for the micropapillary pattern, which displayed moderate agreement.

**Table 6:** The inter-observer concordance rates and the Fleiss’ kappa (κ) statistics for the inter-observer analysis.

	Concordance Rate	Fleiss κ	95% CI
Predominant subtypes	76.1%	0.846	0.843–0.849
L (presence)	86.5%	0.814	0.809–0.819
A (presence)	98.0%	0.949	0.944–0.954
P (presence)	78.0%	0.708	0.703–0.713
S (presence)	92.0%	0.829	0.824–0.834
M (presence)	75.0%	0.531	0.526–0.536
CGP (presence)	88.0%	0.810	0.804–0.815

A, Acinar pattern; CGP, Complex glandular pattern; CI, confidence interval; L, Lepidic pattern; M, Micropapillary pattern; P, Papillary pattern; S, Solid pattern.

## Discussion

The use of intraoperative pathological diagnosis has proven to be an effective method to guide the surgical resection strategy^15^. Recent studies indicated that sublobar resection provided similar clinical outcomes for patients with low-risk pathological subtypes compared to lobectomy^20–22^. Therefore, accurate identification of pathological subtypes is urgently needed. FS, a validated technique for intraoperative evaluation of histological types of lung cancer with feasible precision, emerges as a promising method for guiding surgery by diagnosing pathological subtypes^23–26^. To evaluate the performance of FS in diagnosing the pathological subtypes for early-stage lung adenocarcinoma, we conducted a prospective multicenter study involving 935 cT1N0M0 patients. Our results indicated favorable accuracy of FS in diagnosing pathological subtypes among cT1N0M0 invasive lung adenocarcinoma, particularly excelling in larger lesions and diagnosing the acinar pattern. Our study offers valuable insights into utilizing FS to diagnose pathological subtypes, thereby guiding surgical procedures effectively.

Our study unveiled favorable concordance between intraoperative FS and postoperative FP, aligning with findings from previous studies. Wei *et al*.^27^ conducted a retrospective study evaluating the precision of FS for diagnosing the histological type of lung cancer, revealing an accuracy of 79.8% for the diagnosis of lung adenocarcinoma. FS has demonstrated favorable performance in diagnosing the pathological subtypes of lung adenocarcinoma. Trejo Bittar *et al.* ^28^ retrospectively reviewed 112 patients with stage I adenocarcinoma, revealing favorable sensitivity and specificity of FS in the diagnoses of the pathological subtypes of lung adenocarcinoma, with κ scores ranging from 0.43 to 0.58. Nevertheless, prospective studies addressing this issue remain limited. This study serves as the first prospective clinical trial confirming the favorable concordance between FS and FP.

Intraoperative FS exhibited varying precision in the predominant subtype of lesions, with the highest accuracy for identifying the acinar pattern and the lowest accuracy for the micropapillary pattern. Yeh *et al*.^29^ reviewed 361 resected stage I lung adenocarcinomas with the size of less than 3 cm, observing moderate agreement on the predominant pathological subtype between FS and FP (κ=0.565). Another study also reported that acinar and solid patterns were most likely to be correctly identified by FS, while the micropapillary pattern was not^28^. Zhao *et al.* ^30^ also noted poor accuracy in diagnosing the presence of micropapillary patterns by FS, with a sensitivity ranging from 43.2 to 65.3%. The acceptable performance of FS in diagnosing CGP was noted in Ding’s study, with a moderate diagnostic agreement between FS and FP^31^. Considering these findings, it is crucial to acknowledge that the accuracy of FS varies for different subtypes, which should be considered when applying the results of this study in clinical practice.

The interobserver agreement is another factor affecting the accuracy of FS diagnoses. In our study, 10 pathologists underwent a training session to ensure credibility and reliability. The interobserver consistency assessment was conducted among three pathologists, revealing remarkable agreement. Consistent with our findings, Xu *et al*.’s^32^ study also reported feasible agreement between observers. The study conducted by Ding *et al.* ^31^ demonstrated high interobserver agreement for detecting CGP by FS. In our study, we identified the lowest interobserver agreement in evaluating the presence of the micropapillary pattern. This discrepancy may stem from the relatively lower proportion of micropapillary patterns within early-stage lung cancer lesions. This underscores the importance of further refining diagnostic criteria and ensuring adequate training, particularly for subtypes with low prevalence.

With advancements in precise intraoperative diagnosis, a personalized surgical strategy may become a reality. Previous studies have highlighted that lung cancer patients with low-risk predominant subtypes may be suitable candidates for sublobar resection. For instance, Yao *et al.* ^17^ reviewed 311 patients with subcentimeter lung adenocarcinoma and indicated no significant difference among wedge resection, segmentectomy, and lobectomy in terms of recurrence-free survival and overall survival in patients without the micropapillary pattern. Another study reported that segmentectomy was significantly associated with worse recurrence-free survival and overall survival in patients with micropapillary pattern >5% compared to lobectomy but not in those with micropapillary pattern ≤5%^33^. Precise intraoperative subtype diagnosis plays a crucial role in guiding surgical strategies and identifying candidates for sublobar resection, thereby optimizing treatment outcomes for patients with lung malignancies.

There were several limitations of this study. First, the primary objective of this study is to assess the diagnostic accuracy of FS in identifying pathological subtypes of lung adenocarcinoma. Unfortunately, due to the current unavailability and immaturity of survival data, the impact of different pathological subtypes and surgical procedures on patient survival cannot be examined until several years later. Second, as the study was designed as an observational trial, further studies are warranted to determine the impact of the surgical strategy guided by FS. Third, 10 pathologists participated in the diagnosis of intraoperative FS. The bias might not be completely avoided, although feasible interobserver concordance was revealed.

## Conclusion

In this study, we conducted a large-scale prospective trial validating FS for diagnosing pathological subtypes in cT1N0M0 invasive lung adenocarcinoma. We observed favorable concordance between FS and FP, particularly in patients with larger lesions and APA. A higher rate of misdiagnoses by FS was detected in patients with LPA. The presence of an acinar pattern diagnosed by FS was an independent predictor for the concordance between FS and FP. Overall, our results underscore the significance of FS in diagnosing pathological subtypes, which may help optimize surgical procedures for patients with early-stage lung adenocarcinoma.

## Ethical approval

The protocol was approved by the institutional review board of Fudan University, Shanghai Cancer Center, 270 Dongan Road, Shanghai, China on July 15, 2021 (2021-377-2680). This clinical trial was registered at https://clinicaltrials.gov (NCT05794711).

## Consent

All authors give consent for the publication of the manuscript.

## Sources of funding

This work was supported by the National Natural Science Foundation of China (81930073), the Shanghai Science and Technology Innovation Action Project (20JC1417200), and the Cooperation Project of Conquering Major Diseases in Xuhui District (XHLHGG202101).

## Author contribution

Z.F. and C.D.: performed data curation, methodology, formal analysis, writing – original draft, and writing – review and editing. H.C.: performed validation, data curation. X.S.: performed validation, data curation, and investigation. Q.Z., Y.J., Y.L., Y.Y., C.Y., L.Z., Suying Zuo, S.Y., Q.G., B.Q., W.W., Q.S., J.M., Senzhong Zheng, and G.S.: performed data curation and investigation. Y.Z. and Y.L.: performed conceptualization and supervision. F.F.: performed writing –review and editing, conceptualization, and supervision. H.C.: performed conceptualization, supervision, funding acquisition, and resources. All authors read and approved the final manuscript.

## Conflicts of interest disclosure

The authors declares no conflicts of inter

## Research registration unique identifying number (UIN)

This clinical trial was registered at https://clinicaltrials.gov (NCT05794711).

## Guarantor

Yuan Li, Haiquan Chen, Fangqiu Fu, and Yang Zhang.

## Data availability statement

The data that support the findings of this study are available from the corresponding author upon reasonable request.

## Provenance and peer review

Not commissioned, externally peer-reviewed.

## Supplementary Material
