# Supplementary material for: Prediction of the pathological subtypes by intraoperative frozen section for patients with cT1N0M0 invasive lung adenocarcinoma (ECTOP-1015): a prospective multicenter study
Source: Int J Surg. 2024 May 23;110(9):5444–51. doi: 10.1097/JS9.0000000000001667 (PMC11392073; doi:10.1097/JS9.0000000000001667)
Supplement: Supplementary file 2 [file js9-110-5444-s002.docx]

Supplementary Table 1: The concordance rate between FS and FP among patients with tumor size ＞ 2cm and ≤ 2cm, measured by pathology and radiology, for the diagnosis of predominant pathological subtype

|  | **＞2cm** | | | | | | **≤2cm** | | | | |  |  |
| --- | --- | --- | --- | --- | --- | --- | --- | --- | --- | --- | --- | --- | --- |
|  | **Category** | **Sensitivity** | **Specificity** | **PPV/Precision** | **NPV** | **Accuracy** | **Sensitivity** | **Specificity** | **PPV/Precision** | **NPV** | **Accuracy** | **P(sensitivity)** | **P(specificity)** |
| **Radiology** | **L** | 76.9% | 96.2% | 43.5% | 99.1% | 95.4% | 61.0% | 95.5% | 67.1% | 94.2% | 90.9% | 0.272 | 0.872 |
|  | **A** | 79.2% | 74.5% | 86.0% | 64.6% | 77.7% | 80.6% | 73.3% | 87.4% | 62.4% | 78.5% | 0.706 | 0.505 |
|  | **P** | 71.4% | 90.9% | 65.2% | 93.0% | 87.2% | 72.8% | 89.9% | 51.5% | 95.7% | 87.6% | 0.854 | 0.709 |
|  | **S** | 75.0% | 96.0% | 58.1% | 98.1% | 94.6% | 68.4% | 98.1% | 54.2% | 98.9% | 97.1% | 0.633 | 0.071 |
|  | **M** | 27.2% | 99.7% | 75.0% | 97.7% | 97.4% | 0 | 99.8% | 0 | 99.1% | 99.0% | 0.195 | 0.703 |
| **Pathology** | **L** | 88.9% | 98.0% | 57.1% | 99.7% | 97.7% | 60.4% | 94.5% | 62.0% | 94.1% | 90.1% | 0.094 | **0.042** |
|  | **A** | 80.7% | 80.0% | 89.1% | 67.3% | 80.1% | 79.9% | 70.6% | 85.9% | 61.1% | 77.0% | 0.813 | 0.052 |
|  | **P** | 76.3% | 90.6% | 65.6% | 94.2% | 87.8% | 69.2% | 90.1% | 51.9% | 95.0% | 87.3% | 0.366 | 0.925 |
|  | **S** | 81.8% | 95.5% | 58.0% | 95.6% | 94.5% | 61.9% | 98.2% | 54.1% | 98.7% | 97.0% | 0.146 | **0.020** |
|  | **M** | 33.3% | 99.7% | 75.0% | 98.0% | 97.7% | 0 | 99.8% | 0 | 98.9% | 98.7% | 0.090 | 0.603 |
