# Supplementary material for: Prediction of the pathological subtypes by intraoperative frozen section for patients with cT1N0M0 invasive lung adenocarcinoma (ECTOP-1015): a prospective multicenter study
Source: Int J Surg. 2024 May 23;110(9):5444–51. doi: 10.1097/JS9.0000000000001667 (PMC11392073; doi:10.1097/JS9.0000000000001667)
Supplement: Supplementary file 3 [file js9-110-5444-s003.docx]

**Supplementary Table 2**. Univariate and multivariate analysis for factors associated with discrepancy between FS and FP

|  | Univariate Analysis |  | Multivariate Analysis |  |
| --- | --- | --- | --- | --- |
|  | OR(95%CI) | P value | OR(95%CI) | P value |
| Gender | 0.932(0.679-1.279) | 0.662 |  |  |
| Age |  | 0.997 |  |  |
| Smoking history | 1.061(0.753-1.496) | 0.734 |  |  |
| Lymph node involvement | 0.912(0.630-1.320) | 0.624 |  |  |
| Tumor Size | 0.998(0.975-1.022) | 0.856 |  |  |
| Predominant subtype |  |  |  |  |
| L |  | ref |  |  |
| A | 1.079(0.172-6.751) | 0.935 |  |  |
| P | 0.238(0.039-1.450) | 0.120 |  |  |
| S | 1.133(0.184-6.961) | 0.893 |  |  |
| M | 1.161(0.180-7.511) | 0.875 |  |  |
| Present Subtype (FS) |  |  |  |  |
| L | 1.292(0.937-1.781) | 0.118 |  |  |
| A | **0.288(0.203-0.409)** | **＜0.001** | **0.309(0.216-0.442)** | **＜0.001** |
| P | **1.385(1.015-1.889)** | **0.040** | 1.166(0.843-1.613) | 0.353 |
| S | 1.360(0.916-2.020) | 0.127 |  |  |
| M | 0.891(0.600-1.325) | 0.570 |  |  |
| CGP | **0.622(0.439-0.881)** | **0.007** | 0.309(0.501-1.027) | 0.069 |
| Radiology | 0.974(0.703-1.350) | 0.873 |  |  |
| LVI | 0.675(0.395-1.155) | 0.152 |  |  |
| VPI | 0.780(0.483-1.258) | 0.308 |  |  |
| STAS | 0.775(0.507-1.185) | 0.240 |  |  |
